# Omicron adopts a different strategy from Delta and other variants to adapt to host

**DOI:** 10.1038/s41392-022-00903-5

**Published:** 2022-02-10

**Authors:** Xiaohong Du, Haijun Tang, Long Gao, Zhao Wu, Fang Meng, Ruhong Yan, Shigang Qiao, Jianzhong An, Chen Wang, F. Xiao-Feng Qin

**Affiliations:** 1 Institute of Clinical Medicine Research, Affiliated Suzhou Science and Technology Town Hospital of Nanjing Medical University, Suzhou, 215153 China; 2 Institute of Systems Medicine, Chinese Academy of Medical Sciences & Peking Union Medical College; Suzhou Institute of Systems Medicine, Suzhou, 215123 China; 3 Department of Infectious Disease, The First Affiliated Hospital of Anhui Medical University, Hefei, 230000 China

**Keywords:** Infection, Vaccines, Infectious diseases

**Dear Editor**,

With the ongoing of Corona Virus Disease 2019 (COVID-19) epidemic, new strains of severe acute respiratory syndrome coronavirus 2 (SARS-CoV-2) are continuously emerging. After Delta (B.1.617.2) identified in India, Lambda (C.37), Mu (B.1.621), and Omicron (B.1.1.529) were successively found in Peru, Colombia, and Botswana/South Africa, and subsequently defined as variant of interest (VOI) or variant of concern (VOC). Three VOC strains (Alpha (B.1.1.7), Beta (B.1.351), and Delta) caused the first three waves of global epidemics. However, as the leading variant of the fourth wave of epidemic, the character of Omicron strain is still elusive. Here, we systematically characterized the infectivity, thermal stability, proteolytic activation, entry path, fusogenicity, and immune response of Lambda, Mu, and Omicron variants.

Lambda, Mu, and Omicron strains have several mutations in spike (S) protein, which maybe alter conformation and interaction with its receptor, neutralizing antibodies or other interactors (Supplementary Fig. 1a). To detect the impact of S mutations, we took advantage of vesicular stomatitis virus (VSV)-based pseudovirus, cell–cell fusion, and nuclear factor kappa B (NF-κB) reporter systems. First, the expression of variants S proteins were comparable, but the cleaved bonds (S2) were different (Fig. 1a). Especially, the cleaved bands of Omicron and Alpha were obviously weak in despite of P681H mutation, implying other mutations surrounding Furin cleavage site (e.g., T716I, N679K) regulate the proteolytic cleavage of S protein. Then, we detected the binding affinity of soluble receptor angiotensin-converting enzyme 2 (sACE2) to S protein expressed 293T with flow cytometry. Compared with D614G, Mu, and Omicron S proteins showed enhanced affinity with sACE2 (Supplementary Fig. 1b-d). The cleavage may destabilize the conformation of S trimer and make virions unstable. However, the thermal stability of Omicron was the same as D614G, and Lambda tended to be weaker, while Mu was the opposite (Fig. 1b, c).

**Fig. 1:**
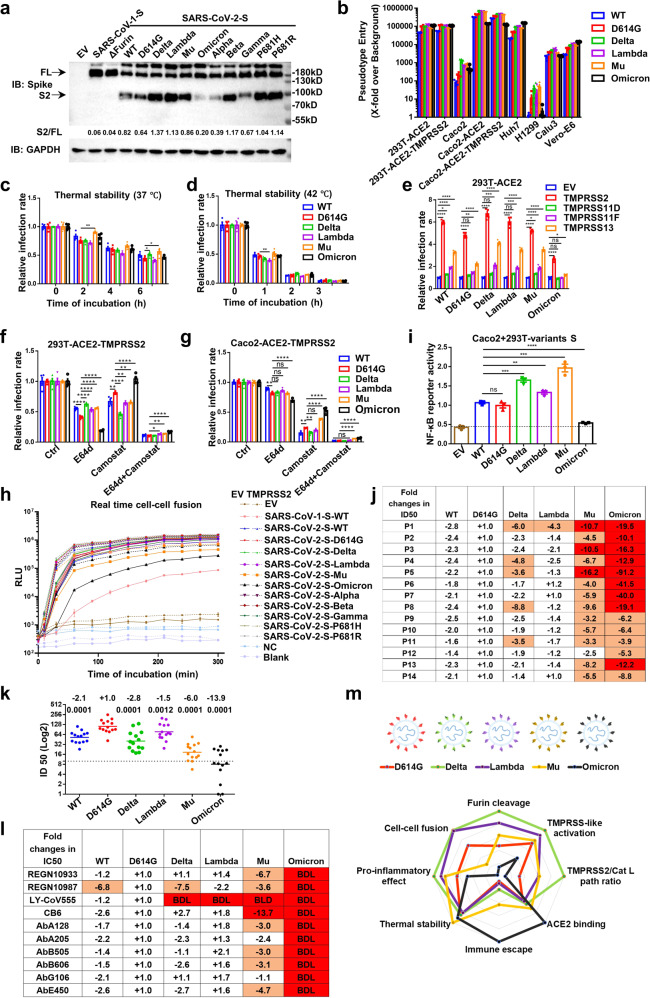
Omicron adopts a different strategy from Delta and other variants to adapt to host. **a** Expression of variants S proteins in 293T cells. **b**, **c** The infectivity of variants pseudovirus in 293T-ACE2 after indicated time of incubation at 37 or 42 °C. **d** The infectivity of variants pseudovirus in several cell lines. **e** The infectivity of variants pseudovirus in 293T-ACE2 transiently overexpressed with TMPRSS2/11D/11F/13. **f**, **g** The infectivity of variants pseudovirus in 293T/Caco2-ACE2-TMPRSS2 cells pretreated with E64d (5 μM) or/and Camostat (50 μM) for 2 h. **h** The dynamic of cell–cell fusion mediated by ACE2 and variants S proteins. **i** The NF-κB reporter activation mediated by incubation with variants S proteins expressing 293T cells. **j**, **k** The neutralization of variants pseudovirus by sera from 14 volunteers received two doses of inactivated SARS-CoV-2 vaccines. The numbers indicate change of geometric mean titers compared with D614G reference virus. **l** The neutralization of variants pseudoviurs by 10 mNAbs targeting RBD of S protein. BDL indicates neutralizing activity below the detection limit when tested at the highest concentration. **m** The radar chart of characters of variants. Experiments were done in 3–4 replicates and repeated at least twice. One representative is shown with error bars indicating SEM

Then, the infectivity of these variants was detected in cell lines with various levels of ACE2 and transmembrane protease serine 2 (TMPRSS2). In general, Delta, Lambda, and Mu pseudovirus showed similar or higher infectivity than D614G. However, the infectivity of Omicron variant was weaker than D614G in Caco2-ACE2/TMPRSS2 and Calu3 cells, but stronger in Caco2, Huh7, and Vero-E6 (Fig. 1d). Moreover, several known membrane-bound serine proteinases (TMPRSS2/11D/11F/13) could significantly promote the infection of all variants, except Omicron (Fig. 1e, Supplementary Fig. 2a, b). Except the entry mediated by TMPRSS-like proteases at cytoplasmic membrane, SARS-CoV-2 also enters via cathepsin at endosomal vesicles. To analyze the selection of entry path, inhibitors (Camostat for TMPRSS-like proteases and E64d for cathepsin B/L) were used to pre-treat the 293T/Caco2-ACE2-TMPRSS2 cells. Compared with D614G, more Delta pseudovirus entered through Camostat-sensitive path, while Omicron was just the opposite (Fig. 1f, g). This variation is probably due to the difference in Furin-related cleavage, deletion of Furin site made most SARS-CoV-2 virus to enter via E64d-sensitive path, and made it like SARS-CoV-1 (Fig. 1a, Supplementary Fig. 2c–e). Moreover, similar with Delta, 12 mutations of Omicron S protein to basic amino acid (K, R, H) generate more amino groups (-NH3+), promoting protonation and sensitivity to endosomal hydrolytic enzymes, such as cathepsin B/L. Overall, Omicron S protein favors endocytic entry path in contrast to Delta and other variants.

Because of the P681R mutation, Delta variant obtains powerful fusogenicity and pathogenicity.^1^ To quantitatively characterize the fusogenicity, the split GFP/renilla luciferase (Rluc8) reporter system was used to monitor the dynamic of cell–cell fusion mediated by S and ACE2. Delta, Lambda, and P681H/R S proteins showed the most powerful fusogenicity, while Mu and Omicron variants were weaker than WT and D614G in despite of P681H mutation (Fig. 1h, Supplementary Fig. 3a, b). Interestingly, Alpha S protein had similar Furin-related cleavage with Omicron, but had similar fusogenicity with WT and D614G, implying some undefined mutations (e.g., N679K, N856K, Q954H, N969K, and L981F) surrounding the S1/S2, S2’ cleavage sites and heptad repeat 1 (HR1) in Omicron S protein regulate the membrane fusion. Moreover, the fusion rate of Omicron was the slowest in all SARS-CoV-2 variants, making it similar with SARS-CoV-1 (Fig. 1h, Supplementary Fig. 3a, b). Data from another cell–cell fusion reporter system mediated by T7 polymerase also demonstrated the similar tendency (Supplementary Fig. 3c, d).

Given the report that the cell–cell fusion mediated by S protein and ACE2 could induce the pyroptosis to exacerbate excessive inflammatory responses,^2^ we speculated that the fusogenicity change could alter the pro-inflammatory effect of Omicron S protein. Reporter assay demonstrated that, compared with WT and D614G, S protein of Delta, Lambda, and Mu variants significantly activated NF-κB pathway (Fig. 1i, Supplementary Fig. 4a, b). Reassuringly, Omicron variant only slightly promoted the activation of NF-κB pathway, which is consistent with early reports that Omicron infected patients and animals show relative mild symptoms.^3,4^ Moreover, the activation of NF-κB pathway was boosted by TMPRSS2, implying the syncytial formation was the leading cause of pro-inflammatory pathway activation (Supplementary Fig. 4c).

Due to the heavy mutation in Omicron S protein, the tendency of immune escape from existed protection of vaccination is worrisome. So, we collected fourteen sera from volunteers who have received two doses of inactivated SARS-CoV-2 vaccines (CoronaVac and BBIBP-CorV) (Supplementary Fig. 5a). Compared with D614G, the neutralizing antibody titer for Lambda, Mu, and Omicron variants decreased 1.5, 6.0, and 13.9 times, respectively (Fig. 1j, k, Supplementary Fig. 5b), explaining the reason why so many confirmed cases have already got fully vaccinated.^3^ Meanwhile, the protection of monoclonal neutralizing antibodies (mNAbs) for Mu and Omicron variants was also destroyed (Fig. 1l, Supplementary Fig. 5c). Alarmingly, Omicron variant fully escaped from the protection of all ten mNAbs targeting receptor-binding domain (RBD), highlighting the importance of implementing strict epidemic prevention policies and developing cocktail therapies and Omicron-specific vaccines.^5^

In this report, we systematically characterized the infectivity, thermal stability, proteolytic activation, entry path, fusogenicity, and immune response of newly emerging SARS-CoV-2 variants, and identified Omicron as a unique strain with different evolutionary strategies from previous strains to adapt to host (Fig. 1m). First, in contrast with Delta and other variants, Omicron favors cathepsin-dependent (E64d-sensitive), but not TMPRSS-like proteases-dependent (Camostat-sensitive) entry path. These findings could explain the change of viral tropism in host tissues and cells with different TMPRSS-like protease level, and suggest combination of TMPRSS-like and cathepsin inhibitors as a reliable treatment for all SARS-CoV-2 variants. Second, in despite of P681H mutation, fusogenicity of Mu and Omicron are significantly weaker than other variants. Third, consistent with fusogenicity, pro-inflammatory effect of Omicron S protein is tempered. Fourth, the heavy mutations give Mu and Omicron variants the strongest ability ever to escape immune protection from vaccination and mNAbs. All these characters of Omicron make it different from Delta and other variants, empowering it to broadly transmit among fully vaccinated population, and changing the tropism and clinical symptoms. To combat against Omicron and other future variants, combination of multiple treatment modalities could be a better and more reliable therapeutic strategy, and variant-specific and pan-β-coronavirus NAbs and vaccines also are the potential prophylactic and therapeutic approaches. Overall, S protein mutations in Lambda, Mu, and Omicron variants alter the infectivity, fusogenicity, and immune response, severely threatening the current therapeutic and prophylaxis approaches, highlighting the importance of implementing strict epidemic prevention policies.

## Supplementary information

Materials and Methods, Supplementary Figure and Figure Legend

## Data Availability

The data that support the findings of this study are available from the corresponding author upon reasonable request.
