# Supplementary material for: Omicron adopts a different strategy from Delta and other variants to adapt to host
Source: Signal Transduct Target Ther. 2022 Feb 10;7:45. doi: 10.1038/s41392-022-00903-5 (PMC8830988; doi:10.1038/s41392-022-00903-5)
Supplement: Supplementary file 1 — Materials and Methods, Supplementary Figure and Figure Legend [file 41392_2022_903_MOESM1_ESM.docx]

**Supplementary Information for**

**Omicron adopts a different strategy from Delta and other variants to adapt to host**

**Authors:** Xiaohong Du^1,2,#,*^, Haijun Tang^2,#^, Long Gao^3,#^, Zhao Wu^2,#^, Fang Meng^2^, Ruhong Yan^1^, Shigang Qiao^1^, Jianzhong An^1^, Chen Wang^1^, F. Xiao-Feng Qin^2,*^

**Materials and Methods**

**RESOURCE AVAILABILITY**

**Lead Contact**

Further information and requests for resources and reagents should be directed to and will be fulfilled by F. Xiao-Feng Qin ([fqin1@foxmail.com](mailto:fqin1@foxmail.com))

**Materials Availability**

Plasmids with SARS-CoV-2 S mutants were generated in our laboratory. SARS-CoV-2 monoclonal neutralizing antibodies are available from AtaGenix Company.

**METHOD DETAILS**

**Cell lines**

Human cell lines (293T, Caco2, Calu3, Huh7 and H1299) and Africa monkey kidney Vero-E6 cell were obtained from American Type Culture Collection. Cells stably expressing human ACE2 and/or TMPRSS2 (293T/Caco2-ACE2/-TMPRSS2) were constructed with lentiviral mediated gene transduction. All above cells were cultured in DMEM (HyClone) or PRMI1640 (HyClone) containing 10% heat-inactivated fetal bovine serum (FBS, Gibco) and 100 U/mL of Penicillin-Streptomycin solution (Gibco). The cells were cultured in an incubator with atmosphere of 5% CO_2_ at 37°C.

**Construction of SARS-CoV-2 variant S protein expression plasmids**

The codon-optimized S gene of SARS-CoV-2 WT (GenBank: MT_613044) was obtained from Genscript Biotechnology Company. To facilitate incorporation of S protein into pseudotyped virons, the last 19 amino acids containing an endoplasmic reticulum (ER)-retention signal in the cytoplasmic tail of the S protein was removed according previously reported ^1^. Variants Alpha, Beta, Gamma, Delta and D614G, P681H and P681R mutations were constructed with ClonExpress Ultra One Step Cloning Kit (Vazyme). Variants Lambda, Mu and Omicron were synthesized at Synbio Technologist Company. All mutations were confirmed with sequencing. The mutation landscape of variants used in this study is illustrated in supplementary Fig. 1a.

**Variants S protein expression and incorporation assay**

To detect the expression of variants S protein, 293T cells were transfected with S protein expression plasmid with lipofectamine 3000 (Invitrogen). 36 h post transfection, cells were lysed by RIPA Lysis Buffer (Beyotime) for 30 min on ice. Then the lysis were centrifugated (12000 rpm for 10 min at 4 °C), and the supernatant was mixed with loading buffer to denature at 100 °C for 10 min. Then samples were separated in a 10% SDS-PAGE, and was blocked by 5% milk for 1 h, and then incubated with primary antibodies overnight and horseradish peroxidase (HRP) conjugated secondary antibodies (1:5000) for 1 h and visualized by Bio-Rad ChgmiDoc MP Bio-Rad Laboratorigs. The following primary antibodies were used: mouse anti-SARS-CoV/SARS-CoV-2 (COVID-19) spike [1A9] (Genetex, 1:2000), mouse anti-VSV matrix protein (Kerafast, 1:2500). Horseradish peroxidase linked anti-mouse IgG antibody (Cell Signaling Technology, 1:5000) was used as a secondary antibody.

**Binding assay of soluble ACE2-hFc with cell surface-expressed variants S proteins by flow cytometry**

293T cells were transfected with variants S protein vectors. After 36-48 h, cells were trypsinized and washed twice with 1 mL staining buffer (PBS with 1% FBS). Then, cells were stained with mouse anti-SARS-CoV/SARS-CoV-2 (COVID-19) spike [1A9] (Genetex, 0.5 μg/mL), or soluble ACE2 recombinant protein (Sino Biological Inc, 0.5 μg/mL) at 4°C for 60 min in 50 μL staining buffer. Then, cells were washed twice with staining buffer and incubated with Alexa Flour488-labeled anti-human IgG Fc (Biolegend, 1:50) or PE-labeled anti-mouse IgG (1:50) at 4°C for 30 min. Then, the cells were washed twice, resuspended and analyzed with Attune NXT flow cytometry (Thermo Fisher Scientific, USA). 293T cells with mock transfection were stained with secondary antibodies as negative control.

**Package and infection of SARS-CoV-2 S protein pseudotyped viruses**

Pseudovirus with SARS-CoV-2 S protein were packaged according reported protocols ^2^. Briefly, 293T cells were transfected with S protein expression plasmid by lipofectamine 3000 (Invitrogen). 12 h after transfection, cells were infected with G*△G-VSV dual reporter virus (VSV G-deleted replication defective recombinant virus containing firefly luciferase and eGFP reporter genes, kindly provided by UltraImmune Inc.). After 6 h of incubation, discarded the supernatant and gently washed the cells twice with PBS, and re-added fresh DMEM medium. 24 h post infection, the supernatant containing the pseudovirions was harvested, then centrifuged at 3500g for 5 min to remove cell debris, and stored at -80°C until use.

To analyze the infectivity of pseudovirus, the susceptible cells were seeded in 96 well and incubated with pseudovirus for 12-16 h, then the cells were lysed for luciferase assay.

**Thermal stability assay of variants pseudovirions**

Pseudovirions incorporated with variants S protein were diluted with DMEM and incubated in a 37°C or 42°C water bath for indicated time, then infected the 293T-ACE2. 12-16 h post infection, the cells were lysed for luciferase assay. The relative infection ratio of pseudovirus was calculated by dividing the relative luminescence unit (RLU) values at each time point by the mean viral RLU at 0 h.

**Split GFP/Rluc8 mediated cell-cell fusion system**

To monitor the cell-cell fusion mediated by ACE2 and S protein in real time, we constructed the split-GFP/Rluc8 reporter system as previously reported ^3^. Briefly, the sequences of spRluc8_155_-spGFP_157_ (spRG) and spGFP_158_-spRluc8_156_ (spGR) were synthesized and inserted into pCAGGS vector. Then, 293T-ACE2 cells (acceptor) were transfected with pCAGGS-spGR, and 293T cells (donor) were transfected with variants S and pCAGGS-spRG. 24 h after transfection, acceptor cells were trypsinized, resuspended at 2 × 10^6^/mL, treated with 60 μM EnduRen™ Live Cell Substrate (Promega) and seeded into 96-Well White Opaque Plates (10^4^ cells/well) to culture at 37 ℃ for 2 h. Then donor cells were resuspended at 2 × 10^6^/mL and mixed with acceptor cells at 1:1 ratio. Then, the mixture was cultured at cell incubator at 37 ℃, and was carried out the luciferase assay at the indicated time.

**T7 polymerase mediated cell-cell fusion system**

We also constructed another reporter system mediated by T7 polymerase to quantitatively detect the fusogenicity of variants S protein. Briefly, we constructed T7-polymerase-T2A-EGFP (T7 pol) and T7 promoter-IRES-luc2 (T7 pro) expression vectors. Then, 293T-ACE2 cells (acceptor) were transfected with T7 pol plasmid, and 293T cells (donor) were transfected with variants S, T7 pro and pRL-TK (as internal control reporter) plasmid. 24 h after transfection, acceptor and donor cells were resuspended at 2 × 10^6^/mL, and mixed at 2:1 ratio. Then the mixture was cultured at 37 ℃ for 12-16 h, photographed for EGFP and lysed for dual-reporter luciferase assay (Promega).

**NF- κB reporter assay**

To analyze the impact of mutations of variants S protein on inflammatory activation of COVID-19 patients, we took advantage the mature NF- κB luciferase reporter. Briefly, Caco2/293T/293T-ACE2 cells (acceptor) were transfected with NF- κB-luc and pRL-TK (as internal control), and 293T cells (donor) were transfected with variants S protein expression vectors. 24 h after transfection, acceptor and donor cells were resuspended at 2 × 10^6^/mL, and mixed at 1:1 ratio. Then the mixture was cultured at 37 ℃ for 12-16 h, and lysed for dual-reporter luciferase assay (Promega).

**Serum samples**

Vaccine sera were obtained from the staff received two doses of CoronaVac and BBIBP-CorV vaccine at Suzhou Science and Technology Town Hospital (Suzhou City, Jiangsu Province, China), stored at -80°C until use. Detailed immunization information is provided in Supplementary Fig. 5a. The study was approved by the Institutional Review Board of Suzhou Science and Technology Town Hospital (IRB2021006). The research was conducted in strict accordance with the rules and regulations of the Chinese government for the protection of human subjects. The study subjects agreed and signed the written informed consents for research use of their serum samples.

**Monoclonal antibodies**

Monoclonal antibodies tested in this study were screened and produced by AtaGenix Company. Antibodies published by other groups including LY-CoV555, CB6, REGN10933 and REGN10987 were synthesized according to the sequences released in Protein Data Bank (PDB). All antibodies were repackaged and stored at -80°C to avoid inconsistent results caused by repeated freeze-thaw cycles.

**Neutralization assays of vaccinee sera and neutralization antibodies**

The neutralization assays of the neutralization antibodies and vaccinated sera were examined as previously reported ^4^. Briefly, serial 3-fold dilution of vaccinee sera (start from 1:10) or serial 5-fold dilution of monoclonal neutralization antibodies (start from 1mg/mL) were incubated with pseudovirions at 37 °C for 1 h, and then infected 293T-ACE2 cells. After 12-16 h, the cells were lysed for luciferase assay. Neutralization activity was defined as the percentage of decrease in luciferase activity compared to the virus control wells (virus + cells). Half-maximal inhibitory concentration (IC50) or half-maximal inhibitory dilution (ID50) was defined as the relative luminescence unit (RLU) values were reduced by 50% compared to the virus control wells (virus + cells) after subtraction of background RLUs in the control groups with cells only. Vaccine sera had neutralizing activity below the detection limit (BDL) even when tested at the highest concentration are assigned a value of 1 for geometric mean calculations and was considered seronegative. The fold changes of each mutant relative to the D614G reference strain in neutralization were calculated by simple division of respective IC50 or ID50 values.

**Statistical Analysis**

The GraphPad Prism 7 software package was used for all statistical analyses. IC50 and ID50 were calculated by the equation of four-parameter dose inhibition response in GraphPad Prism 7. The significance of neutralizing activities of serum samples against each mutant pseudotyped virus relative to D614G was estimated using the Wilcoxon matched-pairs signed rank test. The unpaired two-tails Student’s t-test and one-way ANOVA were used to determine the statistical significance. Experiments were done in 3-4 replicates and repeated at least twice. The values were presented as mean ± SEM and *p* < 0.05 was defined as statistically significant [ns represents no significant difference, *p* < 0.05 (*), *p* < 0.01 (**), *p* < 0.001 (***), *p* < 0.0001 (****)].

**References**

1 Whitt, M. A. Generation of VSV pseudotypes using recombinant ΔG-VSV for studies on virus entry, identification of entry inhibitors, and immune responses to vaccines. *J Virol Methods*. **169**, 365-374, (2010).

2 Li, Q. *et al.* The Impact of Mutations in SARS-CoV-2 Spike on Viral Infectivity and Antigenicity. *Cell*. **182**, 1284-1294.e1289, (2020).

3 Ishikawa, H. *et al.* Generation of a dual-functional split-reporter protein for monitoring membrane fusion using self-associating split GFP. *Protein Eng Des Sel*. **25**, 813-820, (2012).

4 Wang, P. & Nair, M. S. Antibody resistance of SARS-CoV-2 variants B.1.351 and B.1.1.7. **593**, 130-135, (2021).

**Supplementary Figure and Figure Legend**


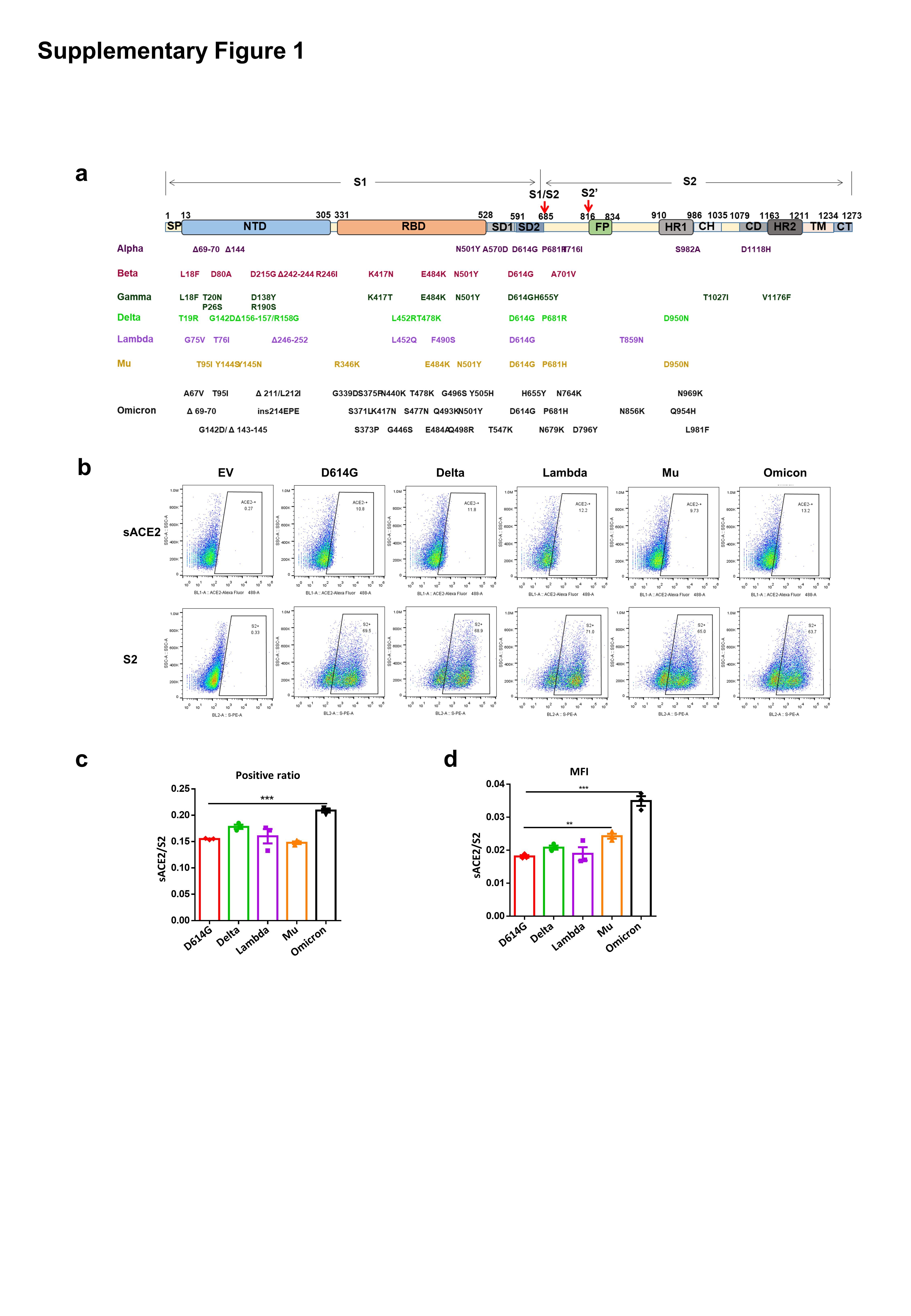


**Supplementary Fig. 1**

**a** Schematic diagram of SARS-CoV-2 variants spike protein mutations. **b-d** The representative image and quantification analysis of binding of soluble ACE2 with cell surface expressed S protein (S2 as internal control).


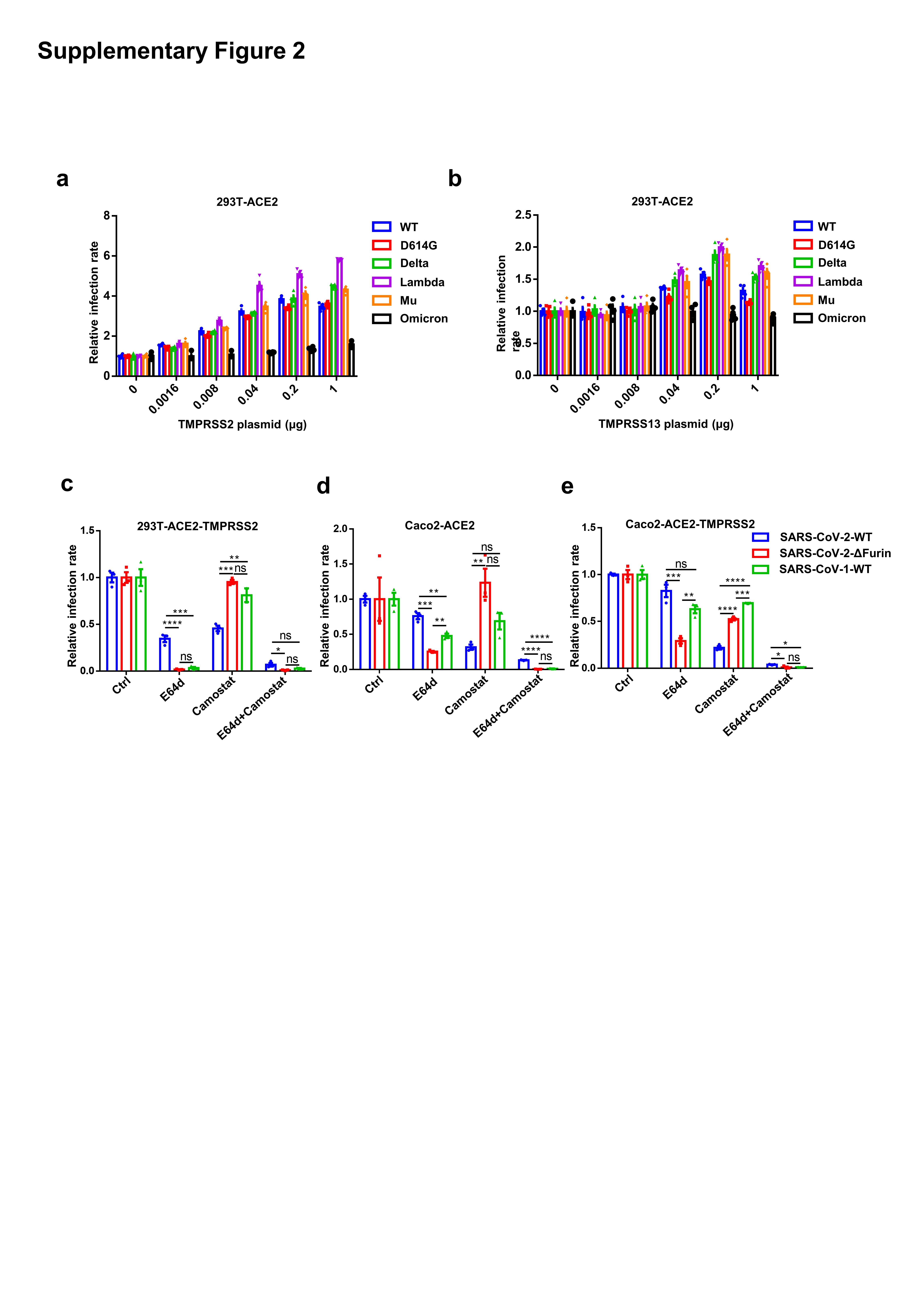


**Supplementary Fig. 2**

**a-b** The infectivity of variants pseudovirus in 293T-ACE2 transiently overexpressed with gradient TMPRSS2/13. **c-e** The infectivity of variants pseudovirus in 293T/Caco2-ACE2/-TMPRSS2 cells pretreated with E64d (5 μM) or/and Camostat (50 μM) for 2h.


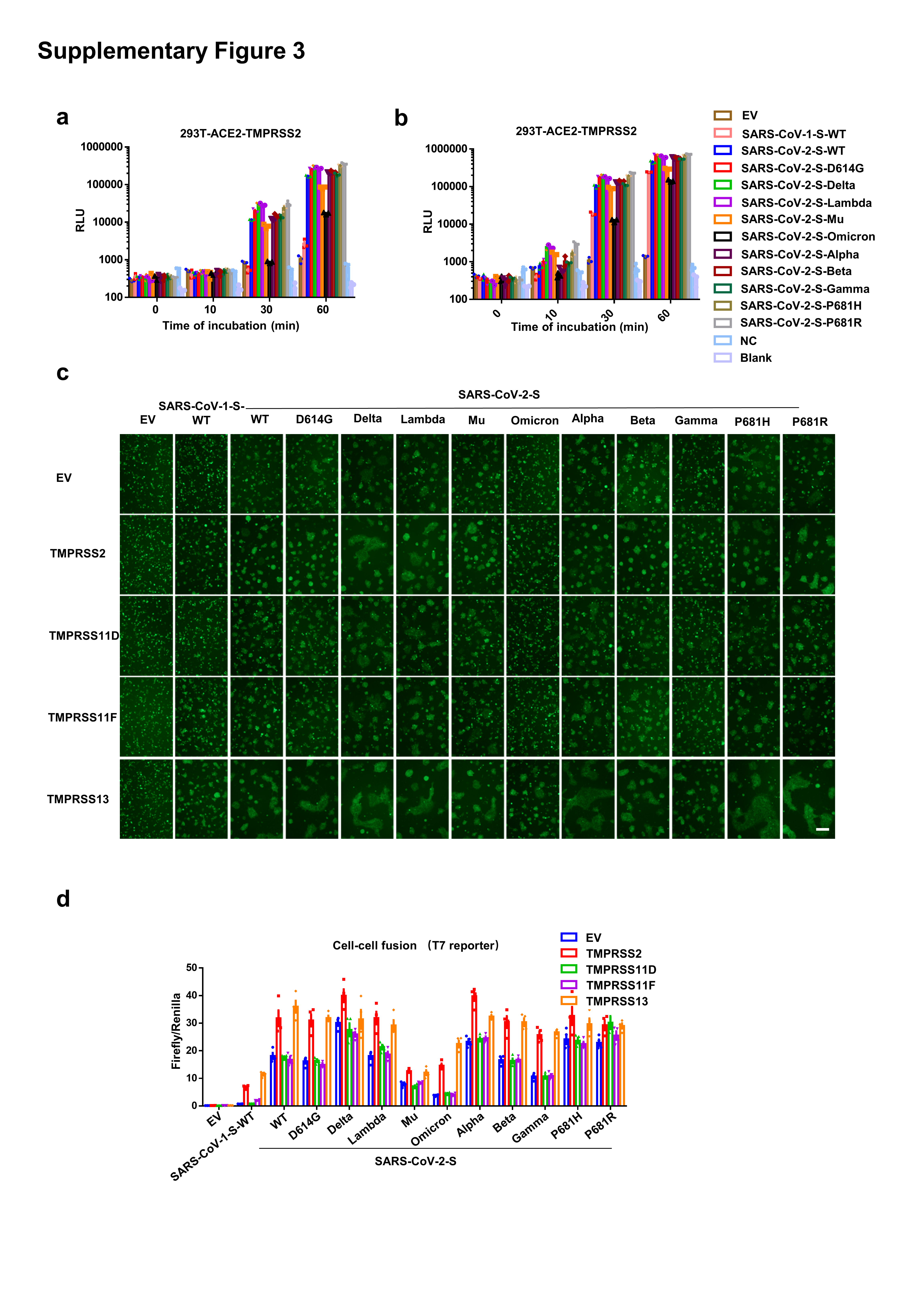


**Supplementary Fig. 3**

**a-b** The dynamic of cell-cell fusion mediated by ACE2 and variants S protein (related to Fig. 1h). **c-d** The representative image and quantification analysis of cell-cell fusion mediated by ACE2/TMPRSS2/11D/11F/13 and variants S protein by T7 polymerase reporter system. The scale bar indicates 200 μm.

**Supplementary Fig. 4**


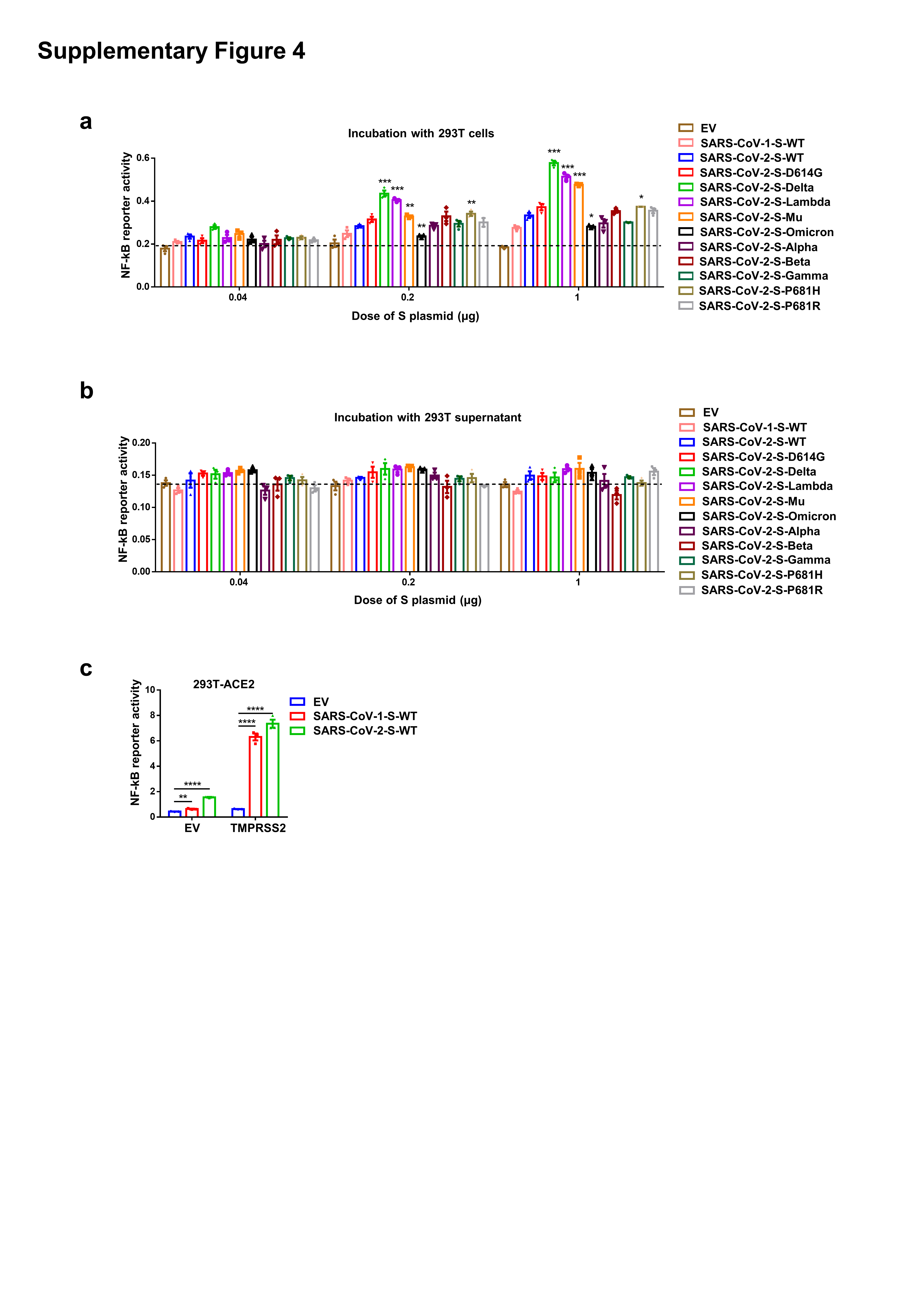


**a-b** The NF-κB reporter activation in Caco2 mediated by incubation with variants S proteins expressing 293T cells or supernatant. The SARS-CoV-2-S-WT was chosen as control for statistical analysis. **c** The NF-κB reporter activation mediated by incubation with variants S proteins expressing 293T cells in 293T-ACE2 transfected with EV or TMPRSS2 vector.


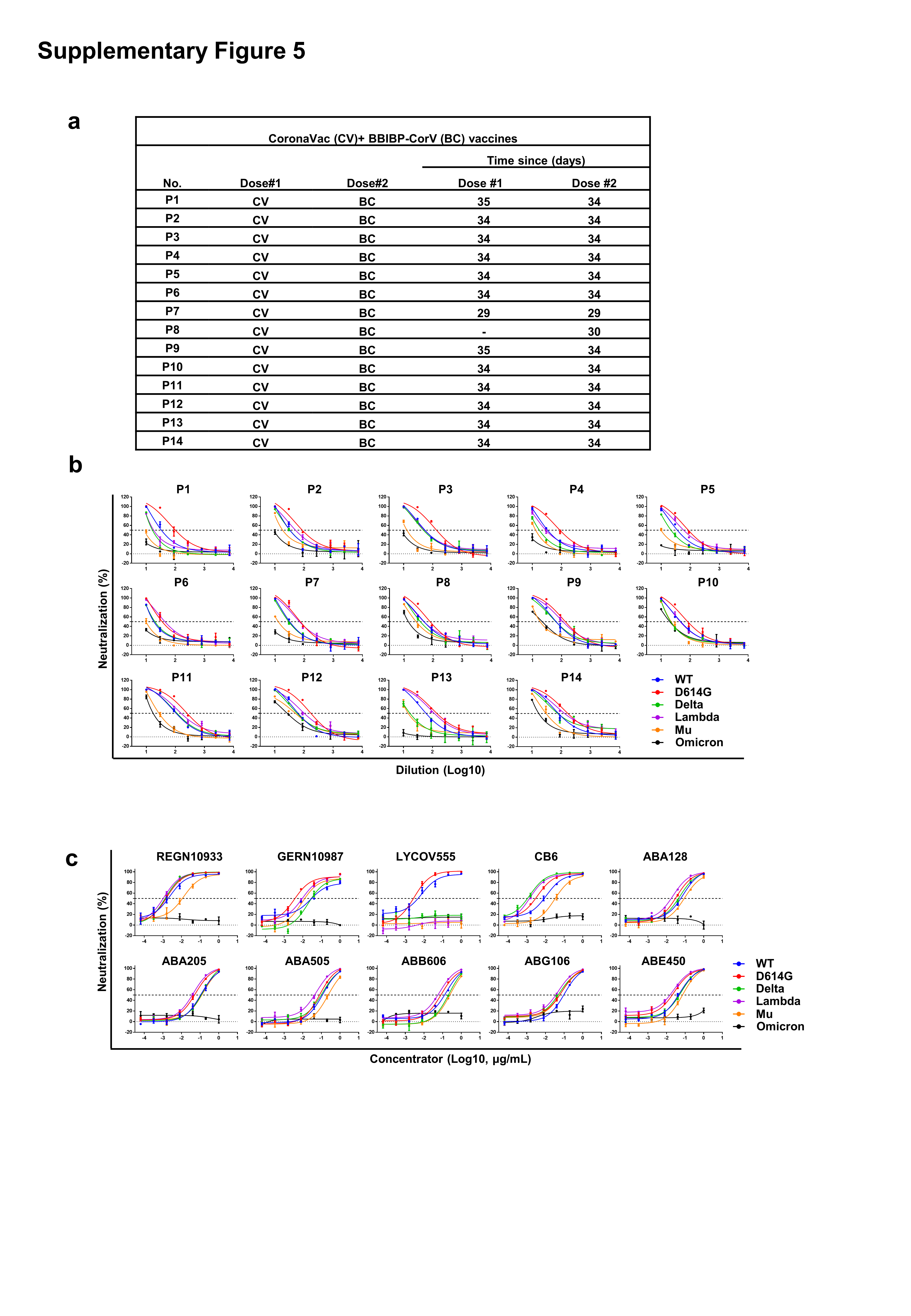


**Supplementary Fig. 5**

**a** Information of the vaccine recipients (related to Fig. 1j, k). **b** Neutralization assay of SARS-CoV-2 variants by vaccination sera (related to Fig. 1j, k). **c** Neutralization assay of SARS-CoV-2 variants by mNAb (related to Fig. 1l).
